# Lipid overload - a culprit for hypertrophic cardiomyopathy?

**DOI:** 10.20517/jca.2022.43

**Published:** 2023-01-01

**Authors:** Lilei Zhang, Na Li

**Affiliations:** 1 Department of Molecular and Human Genetics, Baylor College of Medicine, Houston, TX 77030, USA.; 2 Cardiovascular Research Institute, Baylor College of Medicine, Houston, TX 77030, USA.; 3 Department of Medicine (Section of Cardiovascular Research), Baylor College of Medicine, Houston, TX 77030, USA.

Hypertrophic cardiomyopathy (HCM) is characterized by unexplained left ventricular hypertrophy in the absence of loading conditions such as hypertension or valvular diseases. It is the most common cause of inherited cardiac diseases, with a prevalence of 1 in 500 worldwide^[1]^. It is also the most common cause of sudden death in the young^[2]^. While etiology is heterogenous, at least half of the HCMs with a molecular diagnosis were due to pathogenic or likely pathogenic variants in sarcomere protein encoding genes, predominantly *MYH7* (encoding beta-myosin heavy chain) and *MYBPC3* (encoding cardiac myosin binding protein C)^[2]^. While the pathophysiology of HCM has been heavily debated, the most recent studies converge on the hypercontractility hypothesis^[3]^. Contractile protein mutations that cause either increased contractility or impaired relaxation can result in HCM. The main mechanisms that have been proposed are associated with increased calcium sensitivity, increased myosin head ATPase activity, or reduced myosin super-relaxed state among others. This hypothesis has been well supported by studies utilizing the disease-modeling murine models carrying human pathogenic variants or the patient-derived human induced pluripotent stem cell differentiated cardiomyocytes models^[4]^. The concentric hypertrophy associated with HCM has been attributed to increased calcium induced calcineurin signaling and activation of mitogen-activated protein kinase kinase 1-extracellular signal-regulated kinase 1/2 (MEK1-ERK1/2) signaling pathways^[5]^. In addition to *MYH7* and *MYBPC3* mutations, *TNNI3* (encoding cardiac troponin I, cTnI) is another gene frequently associated with HCM. cTnI is the inhibitory subunit of the troponin complex and serves as a calcium-sensitive molecular switch of the sarcomere contraction in the myocardium. Notably, *TNNI* variants have been reported in 2%–7% of the HCM cases^[6]^. Interestingly, pathogenic variants of *TNNI3* have been associated with HCM, dilated cardiomyopathy, and restrictive cardiomyopathy, presumably due to their differential effect on contractility; however, detailed genotype-phenotype association has not been well established ^[7]^.

*TNNI3* p.R186Q mutation was previously reported in multiple families with HCM and a large Chinese family with co-segregated HCM and atrial fibrillation (AF)^[8,9]^. In this issue of the *Journal of Cardiovascular Aging*, Guo *et al.* characterized the cardiac phenotype in the first *Tnni3*^R186Q/R186Q^ knockin mouse model and investigated the molecular mechanisms underlying the development of HCM^[10]^. Unlike the HCM patients who usually carry heterozygous pathogenic mutation, the heterozygous *Tnni3*^R186Q/+^ mice exhibit relatively normal cardiac function and cardiac morphology. The homozygous *Tnni3*^R186Q/R186Q^ mice exhibit hallmarks of HCM at the age of 10 months including thickening of left ventricular wall, enlarged cardiomyocytes, increased fibrosis, and elevated levels of hypertrophic markers, while the total protein levels of cTnI were unchanged. The overall life span was shorter in *Tnni3*^R186Q/R186Q^ mice than in their wild-type littermates, although the cause of death was not detailed in this study. Utilizing mass spectrometry, they then discovered the differentially expressed proteins in the hearts of *Tnni3*^R186Q/R186Q^ mice compared with the control hearts. Interestingly, enrichment pathway analysis revealed that many altered proteins were involved in fatty acid metabolism. Consistently, they showed that lipid droplets and the levels of total triglycerides and total cholesterol were increased in the hearts of *Tnni3*^R186Q/R186Q^ mice. Because the plasma levels of lipid content remained unchanged, it suggests that the lipid overload in the hearts of *Tnni3*^R186Q/R186Q^ mice is of cardiac origin. Furthermore, fatty acid synthase (FASN) - a key metabolic enzyme involved in the fatty acid metabolism pathway was found to be elevated in the *Tnni3*^R186Q/R186Q^ hearts. They then focused on FASN as an intermediator between aberrant fatty acid metabolism and cardiac hypertrophy [Figure 1]. Strikingly, the dietary intake of the FASN-selective inhibitor C75 for an extended period (i.e., 2 months) improved the cardiac lipid profile, reduced lipid droplets, and attenuated the hypertrophic myopathy in *Tnni3*^R186Q/R186Q^ mice.

Defective lipid metabolism has not been scrutinized in HCM. However, increased contractility and the resulting higher energy demand must be supported by efficient metabolism. Inefficient energy utilization has been shown to cause energetic stress and adverse remodeling^[11]^. Two recent multi-omics studies have shown that energy metabolism is impaired in the heart of HCM patients ^[12,13]^. In particular, increased levels of free fatty acids and reduced acylcarnitines, with broadly downregulated gene expression and protein reduction across fatty acids transport, activation, and β-oxidation, suggest a severely depressed fatty acid metabolism associated with HCM, similar to what have been reported in the advanced heart failure. Inborn errors of fatty acid metabolism and carnitine deficiencies are reported to provoke secondary HCM. The study by Guo *et al.* provides another surprising link between the altered fatty acid metabolism and HCM development^[10]^. The observed lipid accumulation in cTnI R186Q knockin hearts is most likely a result of enhanced fatty acid synthesis, rather than the impaired or slowed breakdown of fatty acids, as the proteins involved in the fatty acid β-oxidation pathway were overall unchanged. Interestingly, human studies on HCM hearts with both sarcomeric and non-sarcomeric variants did not reveal accumulation of triacylglyceride, i.e., lipid droplets^[12,13]^. Further previous animal models, including those with other cTnI variant transgenic models, which recapitulated phenotypic HCM, did not reveal a fatty acid accumulation phenotype^[14]^. Whether the lipid accumulation is unique to the current model or variant or is more broadly applicable requires further evaluation.

The impact of cTnI R186Q mutation on contraction and relaxation of sarcomeres was not directly evaluated in this study. It would be interesting to determine whether the current model represents an example of the hypercontractility hypothesis or an example of a storage disease, such as Fabry disease-associated HCM, which phenocopies sarcomeric HCM. Although the precise molecular mechanism needs to be further elucidated, the study by Guo *et al.* certainly provides new evidence supporting the link between the sarcomeric variant and lipid accumulation. Future investigations should also address whether restoring fatty acid metabolic homeostasis by reducing synthesis or enhancing fatty acid catabolism would be efficacious for HCM. Moreover, considering the recent success of Mavacamten, a cardiac myosin inhibitor for the obstructive HCM^[15]^, it may be worth evaluating whether the patients with the cTnI R186Q mutation might be responsive to the Mavacamten treatment. These potential research questions not only can further provide insights into the molecular mechanisms underlying the cTnI R186Q associated HCM, but also could shed light on the therapeutic indications.

## Figures and Tables

**Figure 1.**
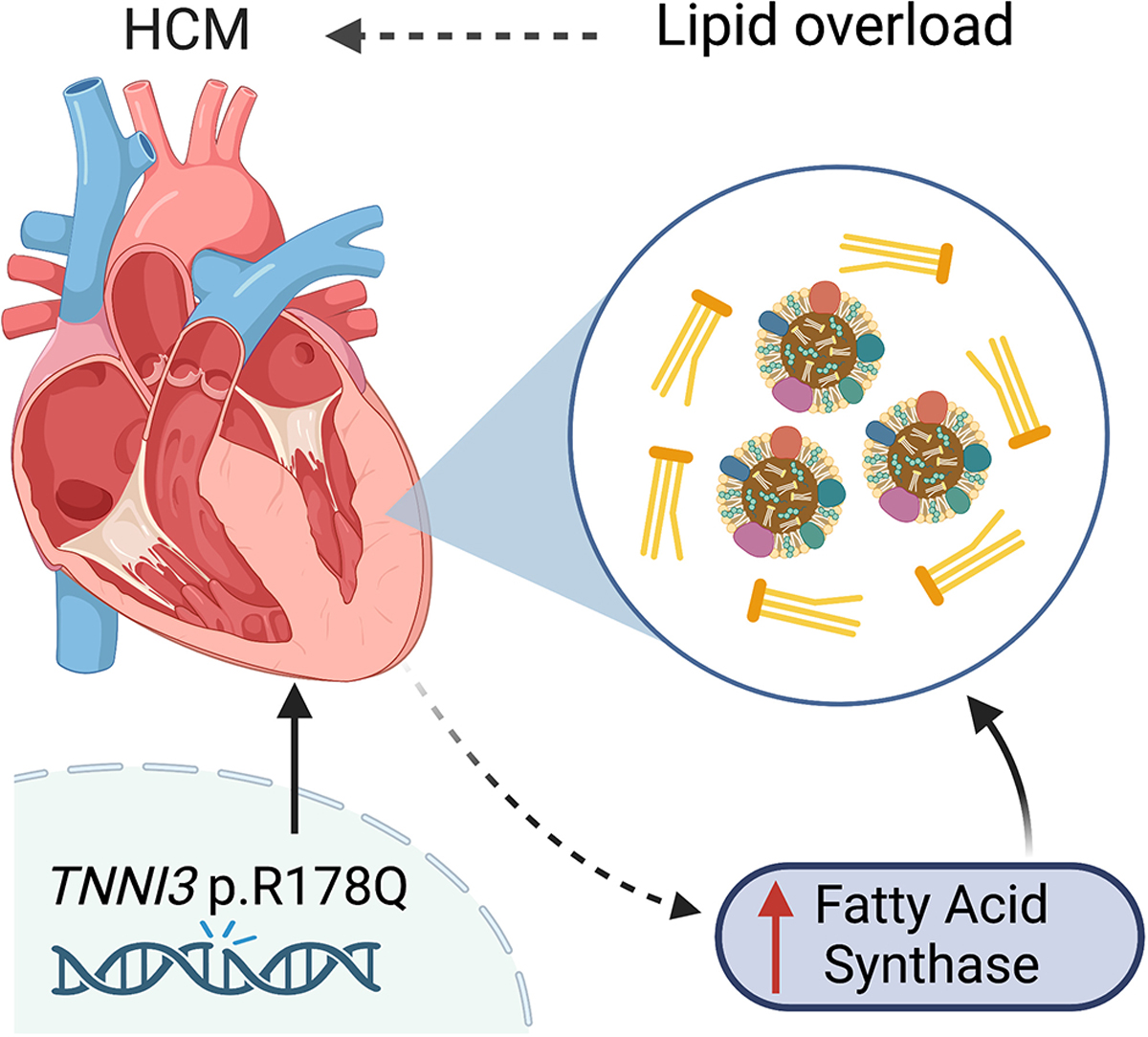
Lipid overload enhances HCM. HCM: Hypertrophic cardiomyopathy.

## Data Availability

Not applicable.
